# Capecitabine Induced Multifocal Leukoencephalopathy: Do We Have Always to Switch off the Chemotherapy?

**DOI:** 10.1155/2016/2408269

**Published:** 2016-02-07

**Authors:** Anastasia Bougea, Panagiota Voskou, Constantinos Kilidireas, Elisabeth Andreadou

**Affiliations:** 1st Department of Neurology, National and Kapodistrian University of Athens Medical School, Eginition Hospital, 74 Vasilissis Sophias Avenue, 11528 Athens, Greece

## Abstract

Capecitabine is a well tolerated and safe 5-fluorouracil agent for adjuvant, neoadjuvant chemotherapy or metastatic cases. Neurological side effects require discontinuation of chemotherapy. We report this unique case of a 50-year-old female, who presented an isolated episode of dysarthria and ataxia under bevacizumab, capecitabine, and oxaliplatin treatment due to reversible multifocal leukoencephalopathy that did not recur after readministration of chemotherapy.

## 1. Introduction

5-Fluorouracil (5-FU)/oxaliplatin-based chemotherapy represents an effective treatment in advanced colorectal cancer, significantly extending progression-free and overall survival rates. Neurotoxicity of 5-FU (capecitabine) has been rarely described as side effect including multifocal leukoencephalopathy particularly in combined regimens with levamisole [1, 2], coma [3], cerebellar syndromes [4], and peripheral neuropathy [5]. However, since chemotherapy-related neurotoxicity is reversible, it remains obscure whether drug reintroduction is beneficial. To the best of our knowledge, only a few cases have been reported to date in literature. We report this unique case that developed 5-FU multifocal leukoencephalopathy as a result of XELOX plus bevacizumab (BV) chemotherapy, with beneficial readministration of lower doses (metronomic chemotherapy).

## 2. Case Presentation

**Figure 1:**
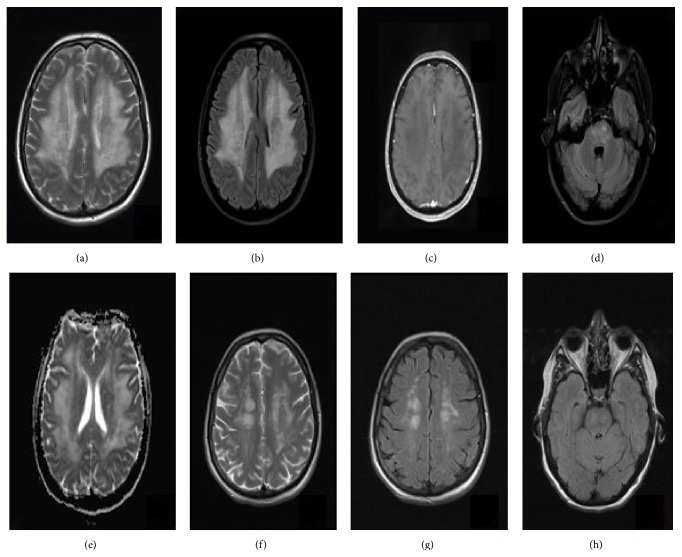
T2-weighted and FLAIR MRI revealed the presence of a high signal intensity mainly in periventricular and subcortical white matter of the bilateral cerebral hemispheres (a, b) and pons (d), without gadolinium enhancement on T1-weighted sequence (c). ADC maps showed increased signal intensity suggestive of vasogenic edema (e). The high signal intensity detected in the deep white matter of bilateral cerebral hemispheres was significantly reduced one year after the onset of symptoms under metronomic chemotherapy ((f) axial T2-sequence; ((g) and (h)) axial FLAIR-sequence).

A 50-year-old female patient with 8-month history of liver and bone metastases from colorectal adenocarcinoma was referred to the Neurological Clinic of University of Athens, because of an episode of dysarthria and ataxia, which lasted two days. She received nine cycles of bevacizumab (Avastin) 7.5 mg/kgr every 21 days, oxaliplatin 130 mg/m^2^ bid every 3 weeks, and capecitabine (Xeloda) 2000/m^2^ bid on days 1–14. On admission, the patient was conscious and well oriented. Complete neurological examination was normal. Blood pressure was within normal range. Mini mental evaluation was 30/30. Laboratory evaluation showed complete blood count, electrolytes, and liver and renal functions within normal limits. Molecular analysis (PCR) for viral etiologies in CSF (cytomegalovirus, varicella-zoster virus, herpes simplex virus type 1 and type 2, Epstein-Barr virus, human herpesvirus 6, and JC) and paraneoplastic markers (antineuronal antibodies: anti-Yo [PCA1], anti-Hu [ANNA1], anti-Ri [ANNA 2], anti-Ma1, anti-Ma2, anti-CV2 [CRMP5], and antiamphiphysin) were normal. Brain magnetic resonance imaging (MRI) revealed multiple symmetric hyperintense lesions in the pons, left splenium of corpus callosum (SCC), bilateral basal ganglia, bilateral thalami, bilateral corona radiata, and bilateral subcortical white matter of parietal lobes on T2-weighted and fluid attenuated inversion recovery (FLAIR) images (Figures 1(a), 1(b), 1(c), and 1(d)). A diagnosis of toxic multifocal leukoencephalopathy was made based on these clinical and radiological findings. Thereafter, chemotherapy was stopped for six months. Α CT scan of the abdomen showed disease progression following a remission interval of one year. Therefore, the patient received capecitabine metronomic chemotherapy (500 mg twice a day continuously). After 3 months of follow-up, the patient was free of neurological signs. Brain MRI at this stage (under metronomic chemotherapy) showed a marked decrease of white matter lesions (Figures 1(f), 1(g), and 1(h)).

## 3. Discussion

Neurotoxicity of 5-FU has been described in patients receiving capecitabine in combination with oxaliplatin such as acute leukoencephalopathy [6] and posterior reversible encephalopathy syndrome (PRES) [7] or with cisplatin in combination with epirubicin [4] or capecitabine with bevacizumab [8]. The present case is unique in uncovering the first patient to develop 5-FU multifocal leukoencephalopathy as a result of XELOX (plus BV) chemotherapy with beneficial reintroduction of lower doses (metronomic chemotherapy).

Nevertheless, it is difficult to prove which chemotherapeutic agent is responsible for the diffuse leukoencephalopathy in our patient, although previous studies indicate that the most common agent is 5-FU [2, 3, 9]. Bevacizumab has been also implicated in the genesis of postchemotherapy leukoencephalopathy, especially PRES. In the literature, however, chemotherapy with multiple agents, rather than with a single drug, is increasingly being associated with leukoencephalopathy [10]. We believe that the combined use of 5-FU and oxaliplatin could have led to more severe and diffuse leukoencephalopathy in our patient.

Differential diagnoses of chemotherapy-related leukoencephalopathy include a variety of conditions that are associated with either the treatment or the disease itself. Brain metastases that could disrupt the blood-brain barrier (BBB) were excluded in our patient. Other uncommon entities, such as paraneoplastic demyelinating disorders that have similar neuroimaging features, were also excluded, as they are not reversible and they worsen without treatment. Moreover, antineuronal antibodies were absent, although this is not a definite exclusion criterion [11]. PRES is characterized clinically by headache, seizures, and altered mental status and radiologically by diffuse hyperintensity in the posterior parietal and occipital white matter, although the grey matter can also occasionally be involved. Although this condition can also be reversible, such a diagnosis was not probable because of the different clinical characteristics and radiological findings [7].

However, little is known about the pathogenetic mechanisms of the chemotherapy-related leukoencephalopathy. Toxicity secondary to antineoplastic agents has been associated with increased permeability of the BBB. Capecitabine intermediate metabolite 50-deoxy-5-fluorouridine (50-DFUR) demonstrates the ability to cross the BBB. Moreover, animals studies showed that 5-FU caused damage to the oligodendrocytes, myelin swelling, and macrophage infiltration resulting in restricted movement of free water [12]. We could hypothesize therefore a direct toxic effect on the endothelium and consequently a temporary damage of the BBB leading to increased permeability of the drug. The hyperintense signal on DWI and the high ADC values in our case suggest vasogenic rather than cytotoxic edema.

Early recognition of chemotherapy-related leukoencephalopathy is important, given that drug discontinuation is generally associated with clinical and radiological improvement. However, spontaneous recovery has been also reported [10]. The benefit of steroids is unclear. It is remarkable that, in a case in which chemotherapy was discontinued due to capecitabine neurotoxicity, no signs were seen after reintroduction of chemotherapy [3]. The natural history and long-term effects remain unknown both in case of continuation or reintroduction of the drug. A good explanation may be the paucity of reported cases of neurotoxicity with capecitabine and the absence of specific prognostic markers for patients at increased risk. An important question remains: can these patients be rechallenged with the same drug? Our case shows that reintroduction of lower doses (metronomic chemotherapy) may be a safe choice. Moreover, our case is noteworthy in that the patient had a good, unusually enduring response to reintroduction of effective chemotherapy, not unusual in other solid tumors [13].

There are not enough data to allow any firm conclusions to be drawn as to whether cerebellar toxicity is more frequent following rechallenge with capecitabine [4]. However, in the absence of an alternative treatment, the drug could be restarted at a lower dose and under close monitoring of the patient. Since the mechanism of action is not fully understood, clinicians should be aware of this rare complication in order to take appropriate therapeutic decisions. Moreover, the choice of timing for administration of monoclonal antibodies (bevacizumab) is significant for achieving the maximum benefit. Furthermore, the selection of appropriate treatment break strategy for patients with advanced cancer is essential. For this purpose, treatment breaks could be incorporated in the treatment plan, as they result in a lower risk of significant toxicity without negative effect in overall survival.
